# Is carbon dioxide a safe and good alternative for diatrizoate meglumine as a contrast in digital subtraction angiography?

**DOI:** 10.4103/0971-4065.50675

**Published:** 2009-01

**Authors:** U. N. Hegde, M. S. Khanapet, M. M. Rajapurkar, S. D. Gang, K. D. Gohel, G. Rane, P. Parikh, D. Patil, T. Desai, P. Patil, N. Kelawala

**Affiliations:** Department of Nephrology, Muljibhai Patel Urological Hospital, Nadiad, India; 1 Department of Surgery and Radiology, College of Veterinary Science and Animal Husbandry, Anand Agricultural University, Anand, Gujarat, India

**Keywords:** CO_2_ angiogram, contrast nephropathy, dog aortogram, enzymuria

## Abstract

Contrast-induced nephropathy is well-known sequelae of iodinated contrast (diatrizoate meglumine). Carbon dioxide (CO_2_) can be used as an alternative contrast agent. The aim of this study was to compare the renal injury and the quality of images of aortogram using iodinated contrast versus CO_2_ using digital subtraction angiography (DSA). This prospective randomized study was done in 29 healthy dogs using DSA aortogram. Dogs were randomly assigned to receive iodinated contrast or CO_2_. 6-F pigtail catheter was introduced via femoral artery approach to perform aortogram under general anesthesia. Serum creatinine (S.Cr.) and urinary enzymes, namely: N-acetyl D-glucosaminidase (NAG), alanine aminopeptidase (AAP), and gamma glutamyl transferase (GGT), were measured before and 48 hours after aortogram. There was no change in S.Cr. in both the groups. Significantly more enzymuria was seen following iodinated contrast than CO_2_. Enzymuria pre and postaortogram following the iodinated contrast was GGT: 14.9 ± 5.92 vs. 26.2 ± 15.1 (*P* = 0.001), NAG: 1.63 ± 0.90 vs. 3.6 ± 2.14 (*P* = 0.0001), and AAP: 1.51 ± 0.75 vs. 3.38 2.41 (*P* = 0.001), and in the CO_2_ group was GGT: 15.5 ± 4.9 vs. 21.1 ± 9.04 (*P* = 0.02), NAG: 2.12 ± 1.06 vs. 3.82 3.27 (*P* = 0.08), and AAP: 1.28 ± 0.76 vs. 2.51 ± 1.72 (*P* = 0.03). More than 50% increase over the preprocedural value was significantly less following CO_2_. Images obtained with iodinated contrast were superior to those with CO_2,_ however, the quality of image with CO_2_ was adequate for delineation of the renal artery and major branches. Both iodinated contrast and CO_2_ cause significant enzymuria. More severe enzymuria (>50% increase) was seen significantly less with the use of CO_2_. Quality of images is better with iodinated contrast.

## Introduction

Angiography using iodinated contrast gives good delineation of anatomy but has the risk of contrast-induced nephropathy (CIN) which has been reported as the third leading cause of acute renal failure in hospitalized patients.[1] The reported incidence of CIN varies from 0[2,3] to 50%[4] due to differences in study design, definition of acute kidney injury, and populations in which it was studied. Carbon dioxide (CO_2_) gas was used in subtraction angiography for the first time[5] in the 1950s.

Carbon dioxide is used as a contrast agent for diagnostic angiography and vascular interventions in both the arterial and venous circulation. When injected into a blood vessel, CO_2_ bubbles displace blood, allowing vascular imaging. Because of the low density of the gas, a digital subtraction angiographic technique is necessary for optimal imaging. CO_2_ is twenty times more soluble than oxygen and dissolves within 2–3 minutes after the injection. It has been reported in several studies that CO_2_ angiography does not affect the renal function.[6,7] Hawkins *et al*, showed that the CO_2_ can cause vapor lock phenomenon in the capillaries and venules, and it can cause renal injury in canine models.[8] Though serum creatinine (S.Cr.) is commonly used as a good measure of renal function, it is a poor marker of early kidney injury. Excretion of tubular enzymes in urine is a sensitive marker of early renal injury.[9,10] As the safety of the CO_2_ is still debatable, we conducted this study to compare the safety and extent of renal injury during aortography, using iodinated contrast versus CO_2_ as a contrast. We compared the renal injury by changes in the S.Cr. and proximal tubular enzymes (N-Acetyl D-glucosaminidase (NAG), alanine aminopeptidase (AAP), and gamma glutamyl transferase (GGT)) and the quality of images of iodinated contrast and CO_2_ using digital subtraction angiography (DSA).

## Materials and Methods

This prospective study was done in 29 apparently healthy dogs between October–December 2003 in collaboration with College of Veterinary science and Animal husbandry, Anand Agricultural University, Anand, after obtaining permission from institutional animal ethics committee and IRB. DSA was used to perform aortogram in dogs. Dogs were randomly assigned to receive iodinated contrast or CO_2_.

After the overnight fasting, all dogs were administered lactated ringer's solution (1 ml/kg body weight I.V.) for six hours prior to anesthesia. Preangiography urine and blood samples were collected before the induction of general anesthesia. The animals in either group were premedicated with acepromazine (0.05 mg/kg body weight I.M.). In all animals induction was done with a mixture of ketamine (10 mg/kg body weight) and diazepam (0.5 mg/kg body weight) as intravenous general anesthetic and maintained using the increments of the same combination.

Arterial access was through the femoral artery. 6F pigtail catheter was introduced via 6F femoral sheath till L1 vertebra. Aortogram was performed using either 2 ml/Kg of diatrizoate meglumine bolus injection or 100 ml CO_2_ using CO_2_ injector. CO_2_ that is 99.99% pure was used. Oxygen saturation levels, blood pressure, and respiratory and heart rates were monitored during the procedure to detect any adverse events. All dogs were observed for seven days for any complications.

Serum creatinine and urinary enzymes (enzymuria), namely NAG, AAP, and GGT, were measured before and 48 hours after the aortogram to assess renal injury. Arterial blood gas analysis was done to measure PaCO_2_ before and immediately after the procedure. The images obtained were compared for the quality and delineation of vascular anatomy.

### Statistical analysis

All the results are presented as mean ± SD or median. Comparisons of the parameters during baseline and at the end of 48 hours were done using paired *t* test. *P*-value less than 0.05 was considered to be statistically significant. The percentage change in the enzymes (GGT, NAG, and AAP) over the preprocedural values was measured by equation: [(Post GGT–Pre GGT)/pre GGT] ×100.

### Laboratory measurements

Safety of the procedure was assessed by the arterial blood gas analysis (Roche omni-C) to check for the accumulation of pCO_2_. The levels of the urinary enzymes were determined by following the method previously reported by us.[11] Ten milliliters of urine was centrifuged at 900 g for 10 minutes. One milliliter of the urine supernatant was loaded on Sephadex G25 column (Bead volume 5.6 ml, previously equilibrated with 0.15 M NaCl solution). The enzymes were eluted out of the column with 2.5 ml of 0.15 M NaCl solution and stored at 0–40°C for analysis. Enzyme activities were measured in a semi-automated spectrophotometer (BT 224, Biotechnica, Italy). GGT was analyzed by following the method of Jung *et al*,[12] whereas NAG and AAP were measured according to the method of Marhun[13] and Jung and Schloz,[14] respectively. The S.Cr. was measured by the Jaffe reaction on a fully automated clinical chemistry analyzer (XL Erba 300 Transasia). Enzyme activities were expressed as units per gram of urinary creatinine. Renal injury was assessed by the renal tubular enzymes (NAG, AAP and GGT) and S.Cr. measurements at baseline and 48 hours after the procedure.

## Results

**Figure 1:**
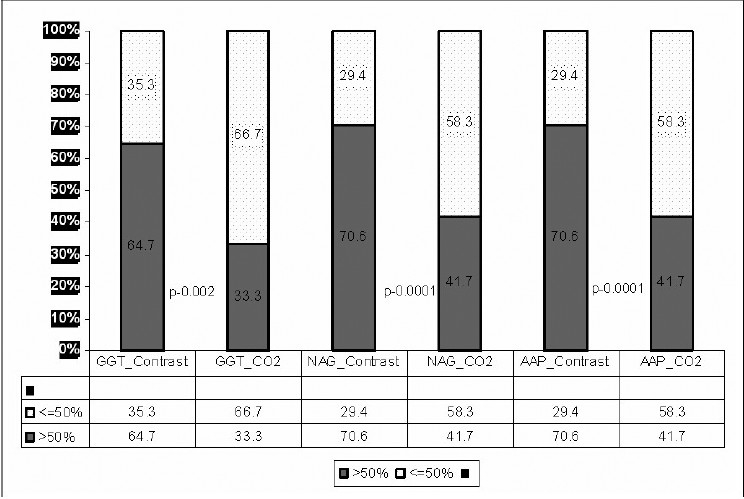
Comparison of percentage change in the tubular enzymes over the preprocedural value between iodinated contrast and CO_2_

Out of 29 dogs that underwent angiography, 12 received CO_2_ and 17 iodinated contrast (diatrizoate meglumine) according to the computer generated randomization. There was no difference in the baseline S.Cr. between the contrast and CO_2_ [Table 1]. There was no difference in the renal function (S.Cr.) in both iodinated and CO_2_ from baseline 48 hours after the aortography. There was increase in the PaCO_2_ after CO_2_ aortography compared to the preaortography level, but it was not statistically significant (*P =* 0.06). The baseline tubular enzymes, namely: GGT (*P* = 0.39), NAG (*P* = 0.09), and AAP (*P* = 0.21), were similar between the contrast and CO_2_. There was significant increase in the enzymuria 48 hours after the aortogram both in the iodinated contrast (GGT: *P* = 0.001; NAG: *P* = 0001; and AAP: *P* = 0.001), and the CO_2_ (GGT: *P* = 0.02; NAG: *P* = 0.08; and AAP: *P* = 0.03) as shown in Table 1. Less than 50 or >50% increase in the enzymes over the preprocedural value is shown in the Figure 1. More than 50% increase was seen with iodinated contrast than CO_2_, GGT: *P* = 0.002; NAG: *P* = 0.0001; and AAP: *P* = 0.0001.

**Table 1:** Comparison of serum creatinine, PaCO2, and urinary tubular enzymes pre and postprocedure

	Iodinated contrast			CO_2_ contrast		
		
	Preangiography	Postangiography (48 hrs)	*P*-value	Preangiography	Postangiography (48 hrs)	*P*-value
PaCO_2_	41.34 ± 4.47	42.31 ± 4.59	0.29	42.03 ± 5.77	45.68 ± 6.56	0.06
S.Cr.	0.81 ± 0.23	0.81 ± 0.25	0.84	0.87 ± 0.2	0.89 ± 0.22	0.69
GGT	14.9 ± 5.92	26.2 ± 15.1	0.001*	15.5 ± 4.9	21.1 ± 9.04	0.02*
NAG	1.63 ± 0.9	3.60 ± 2.14	0001*	2.12 ± 1.06	3.82 ± 3.27	0.08
AAP	1.51 ± 0.75	3.38 ± 2.41	0.001*	1.28 ± 0.76	2.51 ± 1.72	0.03*

* *P*-value < 0.05

**Figure 2:**
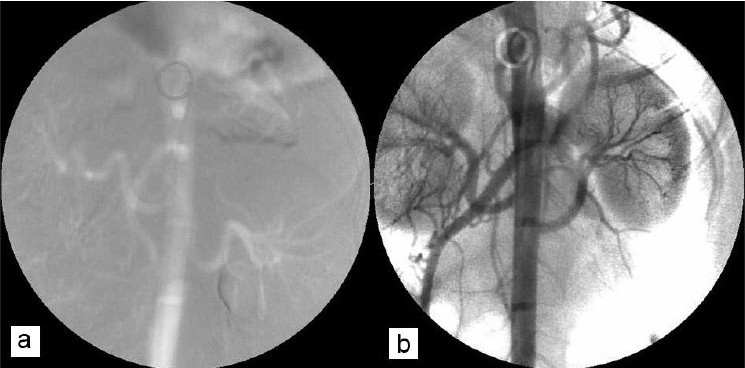
Comparison of DSA images with iodinated contrast (A) and CO_2_ (B)

Images obtained with iodinated contrast are positive images, arterial tree is seen as black, and CO_2_ images are negative images, arterial tree seen as white. Images obtained with iodinated contrast agents [Figure 2a] were superior to those with CO_2_ [Figure 2b]. Main, divisional, and segmental vessels are seen in the iodinated contrast whereas main and divisional vessels are delineated with CO_2_. Although the quality of image with CO_2_ is not as good as that of iodinated contrast, it still gives adequate delineation of the major vascular anatomy.

## Discussion

Hawkins and coworkers explored the use of CO_2_ as an alternative angiographic contrast agent used in combination with DSA. They showed minimal renal toxicity following CO_2_ due to a ‘vapor lock’ phenomenon.[6] Our study focuses on the renal injury measured by tubular enzymes after iodinated contrast and CO_2_. Even though there was no change in the S.Cr, there was significant increase in the tubular enzymes after iodinated contrast and also CO_2_. The degree of increase in the enzymuria was significantly more with the iodinated contrast. This shows that even with CO_2_ there is tubular injury in normal healthy subjects, but significantly less than iodinated contrast.

There are several advantages of the use of CO_2_ instead of conventional iodinated contrast media. Rapid clearance of CO_2_ in lungs prevents recirculation and the renal metabolism is likely to be minimally affected.[7] Iodinated agents, conversely, depend on renal clearance, which results in an increased load in addition to an often-preexisting renal impairment.[15] Another advantage of the use of CO_2_ is that, there is no risk of allergic reactions.[5,16] However, a major drawback of the use of CO_2_ is the lower contrast properties that the gas provides compared with iodinated contrast agents. Another undesirable effect is that some patients experience nausea after CO_2_ injection.[17]

It has been reported in several studies that CO_2_ angiographies does not affect renal function.[6,7] These studies measured only the S.Cr. and did not measure the sensitive markers to show injury or reduction in glomerular filtration rate (GFR). In our study there was no change in the creatinine in both groups but significant increase in the enzymes was observed after the DSA showing tubular injury.

In a recent study, Liss and coworkers[18] found that the risk for impaired renal function in patients after replacement of iodinated contrast medium by CO_2_ is lower than after injection of iodinated contrast medium alone. CO_2_ alone was not sufficient to visualize the small and narrow vessel. PTRA and stent placement could be performed safely with the addition of small dose of iodinated contrast to the CO_2_ without significant deterioration in renal function. Drawback of this study was that it was not possible to totally exclude the use of iodinated contrast medium. Hawkins *et al*, showed that there was a mean decrease in renal blood flow of 11.86% immediately after the CO_2_ injection which returned to baseline after 24 hours in canine model.[8] This study also shows the potential of CO_2_ toxicity, but the intensity is much less than iodinated contrast. The incidence of CIN depends on the renal function,[19] diabetes,[19] osmolality[20] of the contrast agent, and additional nephrotoxic agents. Rudnick *et al*, showed that, in patients without diabetes, whose S.Cr. concentrations were higher than 1.5 mg per deciliter, the incidence of nephropathy was reduced from 27.0 to 12.2% by the use of low-osmolar agent (iohexol) as compared to high-osmolar agent (diatrizoate). Experimental studies in dogs, in which the use of iso-osmolar contrast medium showed no advantage,[20] but in humans the incidence of CIN may be less in high-risk patients when iodixanol (iso-osmolar) is used rather than a low-osmolar nonionic contrast medium.[20]

This study has the following strengths. One, prospective randomized study in canine model, showing the nephrotoxicity and quality of the image. Two, this study measured not only S.Cr. but also the tubular enzymes which are one of the sensitive markers of tubular injury. Arterial blood gas was done to show no significant CO_2_ accumulation. The drawbacks of this study are: one, the study was done in canine model and not in humans. Two, we did not study the effect of CO_2_ with different dosages or multiple boluses. Three, all the dogs were having normal renal functions. Hence, it is difficult to quantify the renal injury with reduced GFR.

The exact mechanism that accounts for the increased risk of CIN has not been determined, but it has been suggested that medullary hypoxia is a crucial factor for the onset of CIN2[1]. *In vitro* conditions were created in which bubbles adhered to the tubing of the circuit, creating functional stenosis, or coalesced into larger bubbles that became trapped, thereby reducing flow and augmenting potential embologenic effects of subsequent injections. In canine models the CO_2_ injections showed potential for ischemic damage owing to vapor lock or transient obstruction to the blood flow in the vessels. The initial lack of blood in the renal cortex after CO_2_ injection can also explain the decrease in GFR. It is highly likely that the filtration of plasma over the glomerular membrane was momentarily stopped when the gas substituted all the blood.

## Conclusion

Both iodinated contrast and CO_2_ cause significant increase in enzymuria. More severe renal injury measured by changes in the tubular enzymes is seen with the iodinated contrast than CO_2_. Quality of images is better with iodinated contrast than CO_2_, but CO_2_ is able to delineate the aorta and main renal artery.
